# Neural correlates of treatment effect and prediction of treatment outcome in patients with PTSD and comorbid personality disorder: study design

**DOI:** 10.1186/s40479-021-00156-8

**Published:** 2021-05-05

**Authors:** Inga Aarts, Chris Vriend, Aishah Snoek, Arne van den End, Matthijs Blankers, Aartjan T. F. Beekman, Jack Dekker, Odile A. van den Heuvel, Kathleen Thomaes

**Affiliations:** 1 Sinai Centrum, Amstelveen, The Netherlands; 2 Department of Anatomy and Neurosciences, Amsterdam Neuroscience, Amsterdam UMC, Vrije Universiteit Amsterdam, De Boelelaan, 1117 Amsterdam, Netherlands; 3 Department of Psychiatry, Amsterdam Neuroscience, Amsterdam UMC, Vrije Universiteit Amsterdam, De Boelelaan, 1117 Amsterdam, Netherlands; 4 Arkin Research, Amsterdam, the Netherlands; 5 Trimbos Institute, Institute of Mental Health and Addiction, Utrecht, the Netherlands; 6 GGZinGeest, Department of Psychiatry, Amsterdam, The Netherlands; 7 VU University, Faculty of Behavioural and Movement Sciences, Amsterdam, The Netherlands

**Keywords:** Borderline personality disorder, Cluster C personality disorder, Treatment, Neuroimaging, Prediction

## Abstract

**Background:**

Neural alterations related to treatment outcome in patients with both post-traumatic stress disorder (PTSD) and comorbid personality disorder are unknown.

Here we describe the protocol for a neuroimaging study of treatment of patients with PTSD and comorbid borderline (BPD) or cluster C (CPD) personality disorder traits. Our specific aims are to 1) investigate treatment-induced neural alterations, 2) predict treatment outcome using structural and functional magnetic resonance imaging (MRI) and 3) study neural alterations associated with BPD and CPD in PTSD patients. We hypothesize that 1) all treatment conditions are associated with normalization of limbic and prefrontal brain activity and hyperconnectivity in resting-state brain networks, with additional normalization of task-related activation in emotion regulation brain areas in the patients who receive trauma-focused therapy and personality disorder treatment; 2) Baseline task-related activation, together with structural brain measures and clinical variables predict treatment outcome; 3) dysfunction in task-related activation and resting-state connectivity of emotion regulation areas is comparable in PTSD patients with BPD or CPD, with a hypoconnected central executive network in patients with PTSD+BPD.

**Methods:**

We aim to include pre- and post-treatment 3 T-MRI scans in 40 patients with PTSD and (sub) clinical comorbid BPD or CPD. With an expected attrition rate of 50%, at least 80 patients will be scanned before treatment. MRI scans for 30 matched healthy controls will additionally be acquired. Patients with PTSD and BPD were randomized to either EMDR-only or EMDR combined with Dialectical Behaviour Therapy. Patients with PTSD and CPD were randomized to Imaginary Rescripting (ImRs) or to ImRs combined with Schema Focused Therapy. The scan protocol consists of a T1-weighted structural scan, resting state fMRI, task-based fMRI during an emotional face task and multi-shell diffusion weighted images. For data analysis, multivariate mixed-models, regression analyses and machine learning models will be used.

**Discussion:**

This study is one of the first to use neuroimaging measures to predict and better understand treatment response in patients with PTSD and comorbid personality disorders. A heterogeneous, naturalistic sample will be included, ensuring generalizability to a broad group of treatment seeking PTSD patients.

**Trial registration:**

Clinical Trials, NCT03833453 & NCT03833531. Retrospectively registered, February 2019.

**Supplementary Information:**

The online version contains supplementary material available at 10.1186/s40479-021-00156-8.

## Introduction

Posttraumatic stress disorder (PTSD) is a common mental disorder with a lifetime prevalence of approximately 4–8% [1–3]. PTSD can develop after experiencing a traumatic event and involves symptoms of re-experiencing, avoidance, hyperarousal and alterations in mood and cognition [4]. Abnormal fear conditioning and extinction learning are important processes in the pathophysiology of PTSD [5]. Psychotherapy for PTSD (trauma-focused therapy, TFT) focuses on normalizing these processes through exposure to the patients’ traumatic memories and trauma-related cues. TFT has moderate to large effect sizes [6, 7]. Previous trials have often excluded comorbidity such as suicidality, so despite the high incidence of comorbidity in PTSD, the efficacy of TFT in this heterogeneous group is not well known. As many as 35% of PTSD patients have a comorbid personality disorder, such as borderline personality disorder (BPD) or avoidant, dependent or obsessive-compulsive (cluster C, CPD) personality disorders [8]. One study has shown that TFT in patients with PTSD and personality disorders (BPD was excluded) is effective [9]. There is also evidence that TFT is effective in patients with PTSD+BPD [10], although a recent meta-analysis shows lower effect sizes for TFT in patients with PTSD and comorbid personality disorders, compared to patients with PTSD-only [11]. Well-established therapies for BPD and CPD are group-based dialectical behavioral therapy (DBT [12]) and schema focused therapy (SFT [13]), respectively.

The amygdala has long been thought to play a crucial role in the pathophysiology of PTSD, because of its involvement in the processing and appraisal of emotional stimuli [14, 15] and its role in the salience network (SN) and limbic network [16]. Although there is some evidence that suggests a smaller volume of the amygdala in PTSD patients [17], other studies found no differences in its morphology compared to healthy controls [18, 19]. In contrast, amygdala volume is consistently lower in BPD patients relative to healthy controls [14], while to the best of our knowledge only one study has been conducted in CPD [20], showing no difference in amygdala volume between patients with avoidant personality disorder and healthy control subjects.

In functional MRI studies, PTSD is associated with hyperreactivity of the amygdala in patients who watch trauma-related stimuli and more generic salient stimuli such as emotional pictures or faces [21, 22]. In BPD, one meta-analysis showed *decreased* activity in the amygdala and associated networks [23], while another meta-analysis [24] showed *increased* left amygdala activity during tasks with a negative emotion versus neutral condition. A possible explanation for these contrasting findings is the heterogeneity of the samples. In line with earlier findings [25], the hyperactivity of the amygdala was not observed in medicated patients [24]. Psychotherapy for PTSD or BPD has been shown to decrease the hyperactivation of the amygdala [26, 27]. This decreased hyperactivation was associated with a decrease in clinical symptoms in PTSD [26] and improved emotion regulation in BPD [28, 29].

Beyond the amygdala, PTSD and personality disorders are also associated with dysfunction of brain areas such as the anterior cingulate cortex (ACC), dorsolateral and dorsomedial prefrontal cortex (dlPFC/dmPFC), insula and the hippocampus (e.g. [30–32]). These regions are all involved in emotion processing and regulation [14, 33], processes that treatments for personality disorders aim to improve [34]. There is some evidence that shows that activity in the above-mentioned brain areas normalizes after treatment. For example, hyperactivation of the ventromedial PFC and ACC normalized after psychotherapy for PTSD [26], and in BPD activation of the ACC decreased after dialectical behavior therapy [27].

Structural and functional connectivity between brain areas is also disrupted in PTSD and personality disorder. In PTSD, as compared to healthy controls, a hyperconnected SN and less well interconnected default mode network (DMN) are found [35–37]. Compared to healthy controls, BPD patients consistently showed hyperactivity of the posterior cingulate cortex (PCC) – a major hub of the DMN – and hypo-activity of the dorsolateral prefrontal cortex (dlPFC) during resting-state functional magnetic resonance imaging (fMRI [23, 24];). BPD patients also showed increased resting state connectivity of the frontopolar cortex and insula [38]. To our knowledge, no neuroimaging connectivity studies have yet been performed in PTSD patients with comorbid personality disorder.

Possible predictors for treatment success in PTSD include clinical features such as the severity of dissociative symptoms [39], baseline PTSD severity and presence of comorbid depressive disorder (see [40, 41] for recent systematic reviews). Previous studies have also shown that neuroimaging measures can be used to predict TFT efficacy, using resting-state functional connectivity measures [42–44], task-related activation patterns [45–49], and regional brain volume [50, 51]. In these studies, the brain areas or networks that are involved in the pathophysiology of PTSD, such as the amygdala, insula and ACC, are also generally the ones that predict TFT outcome. To date, only one study in BPD has reported on treatment outcome prediction, showing that both left amygdala volume and lower amygdala activity during a cognitive reappraisal task associated with better treatment response [52]. No study has yet been performed in patients with CPD.

In the present protocol, we describe a treatment outcome study into the predictive value and pre-post changes of brain function and structure in a comorbid PTSD and personality population (see also [53, 54]). We additionally include a healthy control group for comparison. The first aim is to study the neural alterations induced by PTSD treatment (trauma-focused therapy, TFT) versus integrated PTSD-personality disorder treatment (TFT + PT) in patients with PTSD and comorbid (sub) clinical BPD and/or CPD. Based on previous studies, we expect that 1a) pre-treatment task-related hyperactivity, relative to healthy controls, of the amygdala, ACC and ventromedial PFC will normalize after trauma-focused therapy in both treatment conditions. We further expect that 1b) hyperconnectivity within the SN and hypoconnectivity within the DMN during resting-state normalize towards healthy controls in all patients, regardless of the treatment condition. We additionally expect 1c) more increase in task-related activation in emotion regulation-related areas (e.g. rostral PFC and dlPFC) in patients in the TFT + PT compared to the TFT condition. Finally, we expect 1d) that normalization of brain function is related to improvements in emotion regulation. The second aim is to predict treatment outcome on group and the individual level. Based on previous research, we hypothesize that 2a) a successful treatment outcome can be predicted by lower pretreatment task-related activation of the amygdala, dorsal ACC and insula, 2b) larger volumes of the ACC and hippocampus and 2c) higher severity of PTSD at baseline, higher severity of dissociative symptoms and presence severity of comorbid depressive disorder are related to worse treatment outcomes. Our third and final aim is to compare differences and similarities in the neural alterations in patients with PTSD+BPD and PTSD+CPD, compared to healthy control subjects. We expect 3a) all patients to show pre-treatment dysfunction in task-related activation and resting state connectivity of emotion regulation areas, such as the ACC, insula, dlPFC and hippocampus. In addition, we expect that 3b) in PTSD+BPD patients brain regions of the central executive network (CEN) are hypoconnected. For CPD, these analyses will be more explorative.

## Methods

### Design

**Fig. 1:**
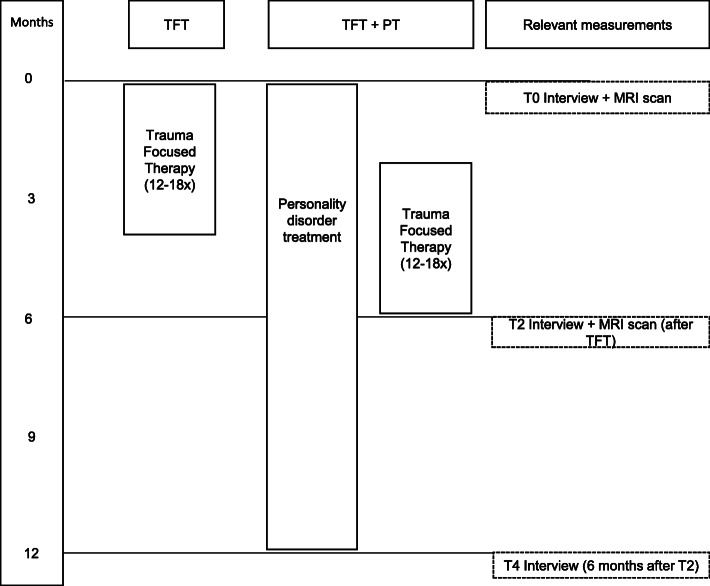
Overview of the timeline for treatment and measurements

This MRI study collects neuroimaging data for patients that participate in the two randomized controlled trials (RCTs) of the PROSPER (Prediction and outcome study in PTSD and personality disorders) study, which is registered under NCT03833453 & NCT03833531 at clinicaltrials.gov [55, 56]. For the MRI study we additionally recruit matched healthy control participants. The study has been approved by the medical ethical committee of the VU University medical center and all participants provide written informed consent in accordance with the Declaration of Helsinki. The PROSPER study consists of two RCTs, with four treatment arms (see Fig. 1). First, patients are divided based on their comorbid personality problems, in either BPD or CPD. Second, they are randomized into the TFT or TFT + PT condition. We use a block-randomization with blocks of six and one random block of four. A researcher not involved in data collection generated the randomization list and an independent person prepared sealed envelopes with assigned treatment condition according to this list. In PTSD+BPD, TFT is eye-movement desensitization and reprocessing (EMDR), TFT + PT consists of EMDR+DBT. In PTSD+CPD, TFT is imaginary rescripting (ImRs), TFT + PT consists of ImRs+SFT. A subset of patients will be asked to additionally participate in the here described add-on MRI study, which involves magnetic resonance imaging (MRI) scan sessions before (T0) and after (T2) TFT in all four conditions. Only the parts of the design relevant to the MRI study will be reported in this paper.

### Sample

We aim to include approximately 80 patients who are enrolled in the PROSPER study, 40 with PTSD+BPD and 40 with PTSD+CPD. We expect to have MRI scans at T2 of at least 40 of these patients (20 BPD and 20 CPD). The inclusion criteria are a primary diagnosis of PTSD and a diagnosis of (sub) clinical BPD or CPD, measured by the SCID-5-PD [57]. We include patients fulfilling at least the required number of SCID-5-PD criteria as defined by the DSM-5 [4] minus one, as to guarantee sufficient generalizability of the population under study. Included patients have an age between 18 and 65 years, have sufficient understanding of the Dutch language and provide written informed consent. Patients are excluded if they have a comorbid disorder that interferes with treatment or randomization; severe outward aggression, addiction or eating disorders interfering with treatment, current psychosis, mental retardation (IQ < 70) or a primary personality disorder diagnosis other than BPD or CPD. Benzodiazepine use exceeding 3 times 10 mg per day oxazepam or equivalent is also an exclusion criterion. Other psychotropic medications need to be stable for at least 3 weeks prior to study participation. For T2 scans, we include patients that have completed their TFT, defined by having received at least 75% of their TFT sessions. Further exclusion criteria related specifically to the MRI study are pregnancy, metal implants, somatic disorders interfering with brain functioning such as head trauma, epilepsy and claustrophobia. Immediately prior to the MRI scan, patients and healthy controls are asked about medication and substance use in the previous 24 h. Use of any benzodiazepines is not allowed in the 24 h before the scan.

We additionally recruit 30 healthy controls through advertisements and Hersenonderzoek.nl (www.hersenonderzoek.nl). The same inclusion and exclusion criteria are used. In addition, healthy controls are excluded if they have a current diagnosis for a mental disorder. This control group will be matched to the patient group average on age, sex and education level.

### Sample size

For reliable fMRI results, a minimum of 20 participants but preferably more than 27 participants has been proposed [58], with more participants a plateau is reached. Furthermore, a minimal sample size of 12 has been proposed with 80% power at α = 0.05 at the single voxel level [59] and we use the same task as a previous study [60] where a robust amygdala activation was found with a sample size of 41 patients. In our study, we include 40 patients with PTSD+BPD and 40 patients with PTSD+CPD. With a predicted dropout of 25–50%, at least 20–30 participants per condition should be available for a second scan. If from the first 80 patients less than 40 return for a second scan, we will continue inclusion until at least 40 post-treatment scans are acquired. Healthy controls will only participate in one scanning session, therefore we include 30 participants.

### Interventions

The treatment protocol is described more detailed in [61, 62], but described here briefly. Patients in the TFT condition will receive EMDR (PTSD+BPD) or ImRs (PTSD+CPD) for a minimum of 12 and a maximum of 18 sessions, delivered within 6 months. TFT sessions are delivered individually, for 75 min weekly. In the TFT + PT condition, patients with PTSD+BPD additionally receive DBT. This consists of six individual pretreatment sessions of 45 min. Patients then start group sessions for 48 weeks, with weekly group sessions of 150 min and biweekly individual 45-min DBT sessions. EMDR starts after 6 weeks of group training.

For patients with PTSD+CPD, the TFT + PT condition consists of ImRs+SFT. For SFT, there are four individual pretreatment sessions of 45 min, after which patients enroll in a group with 90-min weekly sessions for 40 weeks. Additionally, patients receive 18 sessions of group schema focused psychomotor therapy during the course of the SFT-group.

All treatment sessions are recorded on audio (ImRs) or video (EMDR, DBT, SFT) to check treatment adherence. Therapists also fill out a short form about the session, where they note which trauma target was treated and whether there were any protocol violations. Therapists have received accredited training and participate in biweekly supervision meetings. Furthermore, all therapists receive supervision from a trained supervisor. All treatment sessions are recorded on audio (ImRs) or video (EMDR, DBT, SFT) to check treatment adherence. Therapists also fill out a short form about irregularities in the session. Therapists have received accredited training and participate in biweekly intervision meetings. Furthermore, all therapists receive supervision from a trained supervisor.

### MRI acquisition

MRI scans are acquired using a GE Discovery MR750 3-Tesla MRI scanner (General Electric, Milwauki, WI, USA) with a 32-channel head coil at the Amsterdam UMC, location VUmc. We acquire 1) a 3D T1-weighted structural magnetization-prepared 180 degrees radio-frequency pulses and rapid gradient-echo (MP RAGE) according to the ADNI-3 protocol [63], 2) 10 min resting state functional MRI (rsfMRI), 3) fMRI during an emotional faces task, and 4) multi-shell single-spin echo acquisition diffusion weighted images (DWI). Table 1 shows the scan parameters. During the rsfMRI the lights are dimmed and participants are instructed to keep their eyes closed but to not fall asleep. For fMRI and DWI scans, blip-up, blip-down scans with opposite phase encoding directions with the same field-of-view are acquired to correct for susceptibility induced distortions. We use high order shimming to homogenize the B0 magnetic field during the functional scans and the DWI.

**Table 1:** Scan parameters

	3D T1 – MPRAGE	Resting-state fMRI	Task-based fMRI	Multi-shell DWI
TR (ms)	6.9	2200	2200	N/A
TI (ms)	900	N/A	N/A	N/A
TE (ms)	3	28	26	81
Flip angle	9°	80°	80°	90°
# slices	168	42	42	56
# volumes	–	272	205^a^	80
in plane resolution (mm)	1 × 1	3.3 × 3.3	3.3 × 3.3	2.5 × 2.5
Slice thickness	1	3	3	2.5
Slice gap (mm)	N/A	0.3	0.3	N/A
Matrix size	256 × 256	64 × 64	64 × 64	96 × 96
Scan direction	N/A	Axial ascending according to HYFA	Axial ascending according to HYFA	Axial ascending According to HYFA
Shells	N/A	N/A	N/A	73 directions: interleaved 25 b1000, 24 b2000, 24 b3000 + 7 b0

*TR* repetition time; *TE* echo time; *TI* inversion time; *MPRAGE* magnetization-prepared 180 degrees radio-frequency pulses and rapid gradient-echo; *fMRI* functional magnetic resonance imaging; *HYFA* imaginary straight line from the ventral hypophysis to the fastigium in the roof of the fourth ventricle.

^a^ Number of volumes for task-based fMRI varies because the scan is manually stopped around 3 s after the end of the task

### Emotional faces task

In the scanner, patients and healthy controls complete an emotional faces task (EFT) adapted from Frijling [60]. Here we use the conditions: fear, anger and neutral. These face images are contrasted with scrambled faces. Pictures are taken from the Radboud University Database [64]. Three pictures at a time are presented, one at the top and two underneath. For the (emotional) faces, participants have to match the sex of the picture on top to either one on the bottom by clicking a button with their left or right hand. For the scrambled faces, patients have to match the shape of the frame surrounding the scrambled face to the upper picture. Subjects are presented six pseudo randomized blocks of neutral or scrambled faces and five blocks of fearful and angry faces. The same face condition is never presented twice in a row. Each block lasts 20 s (4900 ms stimulus, 100 ms stimulus interval) and contains four different trials of scrambled, neutral or emotional faces. Total task duration is approximately 7 min. We use two counterbalanced versions of the task, one at T0 and one at T2. Pictures are matched on intensity, clarity, attractiveness and valence between the versions. See Supplementary Table 1 for an overview of the models in the task. The images are presented to participants through a projector connected to a computer running E-prime version 2.0.10.353 (Psychology Software Tools, Pittsburgh, PA). Participants see the screen through a mirror mounted on the head coil. For responses, participants press left or right on a button using a Designs Fiber Optic Response Box (FORB) model 932.

### Image processing and quality check

MRI acquisition quality will be checked in several steps. All data will be visually inspected, for example for movement artefacts and ghosting. For fMRI and DWI, the reversed phase-encoded blips to correct for susceptibility-induced distortions are processed using a tool from the FMRIB Software Library (FSL); FSL top-up [65, 66]. To further check quality of T1 and fMRI sequences, the MRI Quality Control Tool (MRI QC [67];) will be used. Quality parameters such as framewise displacement and signal to noise ratio will be calculated for fMRI and DWI sequences and used to assess whether the quality of a scan is acceptable. As an example, scans with a framewise displacement of more than 0.5 mm will be removed before analyses [68].

Structural T1 scans will be processed using FreeSurfer to derive morphometric measures such as volume, surface area and cortical thickness. We will employ the ‘fmriprep’ pipeline to preprocess the fMRI scans [69]; see also below. DWI scans will preprocessed using the FSL EDDY tool [70] to correct for susceptibility-induced distortions, eddy currents and motion. MRtrix3 (www.mrtrix.org [71]) will be used for tractography and DTI-TK [72] for registering the individual DWI scans to a common space to subsequently perform tract-based spatial statistics (TBSS).

### Outcome measures

#### MRI outcome measures

For each MRI modality, we mention the main outcome measures here briefly, they will later be described more fully in pre-registered data analysis plans through the Open Science Framework Project [73].

##### Structural T1 weighted scans

In the structural T1-weighted scans, we will focus mainly on morphometric (cortical thickness, surface area, volume) features of brain areas such as the ACC, insula, hippocampus and amygdala. For the amygdala and hippocampus, we will make sub-segmentations. We will define these brain regions according to standard atlases (e.g. Desikan-Killiany [74] or Brainnetome [75] atlas) and segment these areas on the individual level using FreeSurfer software. Where necessary, we will use voxel-based morphometry for more detailed, parallel analyses.

##### rsfMRI

The main focus of rsfMRI will be on the connectivity of resting-state networks implicated in the pathophysiology of PTSD and personality disorder: the SN, CEN and DMN. Functional connectivity matrices will be made using an atlas-based approach, using standard atlases such as the Brainnetome [75] or Schaefer [76] atlas. This connectivity matrix will then be used to calculate connectivity within and between the three resting-state networks of interest and the connectivity of the amygdala. We will also use graph measures calculated from this matrix to study changes in the topology of the brain network, including global efficiency, clustering coefficient and modularity (see for example [77, 78]).

##### fMRI during emotional face task

In the fMRI during the emotional face task, the main contrast of interest is the fearful faces versus scrambled faces. In addition, we will study the angry versus scrambled faces and neutral versus scrambled faces contrasts as secondary analyses. Our regions of interest include the amygdala, dorsal ACC, anterior insula, vmPFC, dlPFC, dmPFC, hippocampus and visual areas. We will specify the exact coordinates for the regions of interest in the preregistered data analysis plans. For additional connectivity analyses, we will use a generalized form of context-dependent psychophysiological interactions (gPPI [79];).

##### DWI

Integrity of white matter fiber bundles will be measured using standard diffusivity measures: fractional anisotropy (FA), mean diffusivity (MD), radial diffusivity (RD), and axial diffusivity (AD) using DTITK [72] and FSL TBSS. In addition, we will perform whole brain tractography using MRTRIX3 to study between-group differences and treatment-induced changes in structural connectivity and characteristics of the structural connectome using graph measures.

### Clinical outcome

The main clinical outcome measure is the clinician-administered PTSD scale for DSM-5 (CAPS-5, [80]). The CAPS-5 is administered before treatment (T0), directly after TFT (T2) and 6 months after the TFT ends (T4; see Fig. 1 for overview) by trained independent assessors, blind to treatment condition. For this study, the PTSD symptom change between T0 and T4 as measured by the CAPS-5 will be calculated to see whether our imaging outcome measures correlate with and predict clinical improvement. For responder/non-responder analyses, responders will be classified as patients who show an improvement of ≥0.5 standard deviation in pre- to post-treatment CAPS-5 scores [81].

### Statistical analysis

#### Treatment effect

To test hypotheses 1a-1c and analyze treatment effect, we will use linear mixed-models with the above-described MRI outcome measures as dependent variables. For hypothesis 1a, this means amygdala, ACC and vmPFC activation. For 1b, connectivity in resting-state networks and for 1c, activation in emotion regulation areas (such as the rostral PFC and dlPFC). For hypothesis 1d, we will correlate changes in brain activation (from hypotheses 1a-1c) with change in emotion regulation, as measured with change in the Difficulties in Emotion Regulation Scale (DERS [82];) from T0 to T2. We are interested in the main effect of time (T0, T2) and the interaction effect of time x type of treatment (TFT or TFT + PT). The value of the outcome measure at T0 will be added as covariate and a random factor for participants will be added to the model. Additionally, we will make a model with PTSD and personality disorder severity (measured with the CAPS-5 and SCID-5-PD respectively) at baseline, age and sex as covariates. For exploratory analyses, we will compare differences in neural response in treatment responders versus non-responders.

#### Prediction of treatment effect

To predict treatment outcome at T4 (hypotheses 2a-2c), we use CAPS-5 score at T4 as a continuous dependent variable in a multiple linear regression model with MRI outcomes at T0 as independent variables. For hypothesis 2a, task-related amygdala, dACC and insula activation will be added to the model. For hypothesis 2b, volumes of the ACC and hippocampus will be added to the model. For hypothesis 2c, PTSD severity, presence and severity of depressive disorder and dissociative symptoms will be added to the model. Medication use will be used as a covariate.

To predict response versus non-response, we will use logistic regression. Dichotomous treatment response status is the dependent variable, with the predictors mentioned above as independent variables.

We will also conduct more exploratory prediction analyses, using features derived from the different MRI modalities, e.g. resting-state network measures, whole brain activation during the task and DTI-derived measures such as FA and structural connectivity. We will preregister our analysis plan for these analyses prior to data analysis [73].

Furthermore, we will perform sensitivity analyses for patients who use psychotropic medication versus patients who do not use medication, for patients with and without comorbid depression and patients with and without the dissociative subtype of PTSD (measured with the CAPS-5) by running the analyses with and without these groups.

#### Comparison BPD, CPD and healthy controls

To assess differences in brain activation (hypotheses 3a-3b), connectivity and brain morphology between patients with PTSD+BPD, patients with PTSD+CPD and healthy controls, we will use a regression model with diagnosis as independent variable and imaging outcome measures as dependent variables. For hypothesis 3a, task-related activation and resting state activation of emotion regulation areas will be added to the model, for hypothesis 3b, connectivity in resting-state networks will be added to the model. If possible, we will do the analyses separately for medicated and non-medicated patients. We will also analyze the difference in clinical comorbidity and its relation to neuroimaging measures between the groups.

## Discussion

The here described neuroimaging study aims to investigate the neural effects of TFT versus TFT + PT in PTSD patients with a personality disorder, as well as the utility of neuroimaging measures to predict treatment outcome and to study similarities and differences between patients with PTSD and BPD or CPD. This study can provide important insight into a patient group that has not received extensive study until now. We include a heterogeneous group of PTSD patients with (sub) clinical personality disorders, which makes our study generalizable to a broad patient population that is characteristic of the clinical population, since as many as 35% of PTSD patients have a comorbid personality disorder [8].

A downside of this naturalistic approach within a heterogeneous sample, is that there may be confounding factors in interpreting the results. One of these is medication use. While other studies often exclude patients that use medication, patients in our sample use medication that has been stable for at least 3 weeks. This medication may influence structural and functional characteristics of the patients’ brains. Schulze [24] found in their meta-analysis that unmedicated patients show hyperactivation of the amygdala compared to healthy controls, while medicated patients do not. Since lower amygdala activity is expected to predict successful treatment outcome, it is possible that medicated patients react better to treatment. It is also possible that the effects of our TFT or TFT + PT on the brain differs between medicated and unmedicated patients, which we plan to study by running our analyses with and without medicated patients.

Another source of heterogeneity is the presence of other comorbid disorders that may influence our results, such as major depressive disorder (MDD). A meta-analysis showed that 52% of PTSD patients also meet criteria for MDD [83]. PTSD patients with MDD and other comorbid disorders may differ in their underlying neural characteristics. PTSD and BPD are for example associated with a hyperactive amygdala during an emotional task compared with healthy subjects, while MDD is associated with hypoactivation of the amygdala [84]. Secondly, some PTSD patients suffer from dissociative symptoms [4]. Dissociation is related to increased inhibition of the limbic response by areas such as the dorsal ACC and mPFC [39]. Finally, most research in personality disorders has been done in BPD, and data are sparse in the three personality disorders that make up CPD [85]. Different neural mechanisms could underlie these different disorders, but this is hitherto unknown. Where possible, we intend to study patients with and without these comorbid conditions separately to disentangle the confounding comorbidity.

## Conclusion

In conclusion, this neuroimaging study is the first to study neural correlates of treatment effects in patients with PTSD and comorbid personality disorder and imaging-derived biomarkers predicting response. We also aim to study the different underlying neural mechanisms in PTSD patients with BPD and CPD. With this study, we hope to shed light on a population that has so far received little attention in neuroimaging research.

## Supplementary Information

**Additional file 1 Table S1.** Overview of models used in the Emotional Faces task.

## Data Availability

Not applicable, data is not available yet.

## Abbreviations

ACC: Anterior cingulate cortex
AD: Axial diffusivity
BPD: Borderline personality disorder
CEN: Central executive network
CPD: Cluster C personality disorder, i.e. avoidant, dependent and obsessive-compulsive disorder
DBT: Dialectical behavior therapy
dlPFC: Dorsolateral prefrontal cortex
DMN: Default-mode network
dmPFC: Dorsomedial prefrontal cortex
DTITK: Diffusion tensor imaging ToolKit
DWI: Diffusion weighted images
EFT: Emotional faces task
EMDR: Eye-movement desensitization and reprocessing
FA: Fractional anisotropy
fMRI: Functional magnetic resonance imaging
FSL: FMRIB Software Library
gPPI: Generalized form of psychophysiological interaction
HYFA: Imaginary straight line from ventral hypophysis to the fastigium in the roof of the 4th ventricle
ImRs: Imaginary rescripting
MD: Mean diffusivity
MRI: Magnetic resonance imaging
MRIQC: MRI quality control tool
PCC: Posterior cingulate cortex
PFC: Prefrontal cortex
PTSD: Posttraumatic stress disorder
RD: Radial diffusivity
SFT: Schema focused therapy
SN: Salience network
TBSS: Tract-based spatial statistics
TFT: Trauma-focused therapy
TFT + PT: Trauma-focused therapy with personality treatment

## References

[1] de Vries G-J, Olff M (2009). The lifetime prevalence of traumatic events and posttraumatic stress disorder in the Netherlands. J Trauma Stress.

[2] Kilpatrick DG, Resnick HS, Milanak ME, Miller MW, Keyes KM, Friedman MJ (2013). National estimates of exposure to traumatic events and PTSD prevalence using DSM-IV and DSM-5 criteria. J Trauma Stress.

[3] Koenen KC, Ratanatharathorn A, Ng L, McLaughlin KA, Bromet EJ, Stein DJ (2017). Posttraumatic stress disorder in the world mental health surveys. Psychol Med.

[4] American Psychiatric Association (2013). Diagnostic and statistical manual of mental disorders.

[5] Zuj DV, Norrholm SD (2019). The clinical applications and practical relevance of human conditioning paradigms for posttraumatic stress disorder. Prog Neuro-Psychopharmacol Biol Psychiatry.

[6] Watts BV, Schnurr PP, Mayo L, Young-Xu Y, Weeks WB, Friedman MJ (2013). Meta-analysis of the efficacy of treatments for posttraumatic stress disorder. J Clin Psychiatry.

[7] Cusack K, Jonas DE, Forneris CA, Wines C, Sonis J, Middleton JC, Feltner C, Brownley KA, Olmsted KR, Greenblatt A, Weil A, Gaynes BN (2016). Psychological treatments for adults with posttraumatic stress disorder: a systematic review and meta-analysis. Clin Psychol Rev.

[8] Friborg O, Martinussen M, Kaiser S, Øvergård KT, Rosenvinge JH (2013). Comorbidity of personality disorders in anxiety disorders: a meta-analysis of 30 years of research. J Affect Disord.

[9] Markowitz JC, Petkova E, Biyanova T, Ding K, Suh EJ, Neria Y (2015). Exploring personality diagnosis stability following acute psychotherapy for chronic posttraumatic stress disorder. Depression and anxiety.

[10] Slotema CW, Wilhelmus B, Arends LR, Franken IH (2020). Psychotherapy for posttraumatic stress disorder in patients with borderline personality disorder: a systematic review and meta-analysis of its efficacy and safety. Eur J Psychotraumatol.

[11] Snoek A, Nederstigt J, Ciharova M, Sijbrandij M, Lok A, Cuijpers P, et al. Impact of comorbid personality disorders on psychotherapy for post-traumatic stress disorder: systematic review and meta-analysis. Under review. 10.1080/20008198.2021.1929753 PMC8221135 34211638

[12] Stoffers-Winterling JM, Völlm BA, Rücker G, Timmer A, Huband N, Lieb K. Psychological therapies for people with borderline personality disorder. Cochrane Database Syst Rev. 2012;8. 10.1002/14651858.CD005652.pub2 PMC6481907 22895952

[13] Bamelis LL, Evers SM, Spinhoven P, Arntz A (2014). Results of a multicenter randomized controlled trial of the clinical effectiveness of schema therapy for personality disorders. Am J Psychiatr.

[14] Phillips ML, Drevets WC, Rauch SL, Lane R (2003). Neurobiology of emotion perception I: the neural basis of normal emotion perception. Biol Psychiatry.

[15] Etkin A, Büchel C, Gross JJ (2015). The neural bases of emotion regulation. Nat Rev Neurosci.

[16] Seeley WW, Menon V, Schatzberg AF, Keller J, Glover GH, Kenna H, Reiss AL, Greicius MD (2007). Dissociable intrinsic connectivity networks for salience processing and executive control. J Neurosci.

[17] Logue MW, van Rooij SJ, Dennis EL, Davis SL, Hayes JP, Stevens JS (2018). Smaller hippocampal volume in posttraumatic stress disorder: a multisite ENIGMA-PGC study: subcortical volumetry results from posttraumatic stress disorder consortia. Biol Psychiatry.

[18] Kühn S, Gallinat J (2013). Gray matter correlates of posttraumatic stress disorder: a quantitative meta-analysis. Biol Psychiatry.

[19] O'Doherty DCM, Chitty KM, Saddiqui S, Bennett MR, Lagopoulos J (2015). A systematic review and meta-analysis of magnetic resonance imaging measurement of structural volumes in posttraumatic stress disorder. Psychiatry Res Neuroimaging.

[20] Denny BT, Fan J, Liu X, Guerreri S, Mayson SJ, Rimsky L, McMaster A, Alexander H, New AS, Goodman M, Perez-Rodriguez M, Siever LJ, Koenigsberg HW (2016). Brain structural anomalies in borderline and avoidant personality disorder patients and their associations with disorder-specific symptoms. J Affect Disord.

[21] Pitman RK, Rasmusson AM, Koenen KC, Shin LM, Orr SP, Gilbertson MW (2012). Biological studies of post-traumatic stress disorder. Nat Rev Neurosci.

[22] Shin LM, Rauch SL, Pitman RK (2006). Amygdala, medial prefrontal cortex, and hippocampal function in PTSD. Ann N Y Acad Sci.

[23] Ruocco AC, Amirthavasagam S, Choi-Kain LW, McMain SF (2013). Neural correlates of negative emotionality in borderline personality disorder: an activation-likelihood-estimation meta-analysis. Biol Psychiatry.

[24] Schulze L, Schmahl C, Niedtfeld I (2016). Neural correlates of disturbed emotion processing in borderline personality disorder: a multimodal meta-analysis. Biol Psychiatry.

[25] Ma Y (2015). Neuropsychological mechanism underlying antidepressant effect: a systematic meta-analysis. Mol Psychiatry.

[26] Thomaes K, Dorrepaal E, Draijer N, Jansma EP, Veltman DJ, van Balkom AJ (2014). Can pharmacological and psychological treatment change brain structure and function in PTSD? A systematic review. J Psychiatr Res.

[27] Marceau EM, Meuldijk D, Townsend ML, Solowij N, Grenyer BF (2018). Biomarker correlates of psychotherapy outcomes in borderline personality disorder: a systematic review. Neurosci Biobehav Rev.

[28] Goodman M, Carpenter D, Tang CY, Goldstein KE, Avedon J, Fernandez N, Mascitelli KA, Blair NJ, New AS, Triebwasser J, Siever LJ, Hazlett EA (2014). Dialectical behavior therapy alters emotion regulation and amygdala activity in patients with borderline personality disorder. J Psychiatr Res.

[29] Schmitt R, Winter D, Niedtfeld I, Herpertz SC, Schmahl C (2016). Effects of psychotherapy on neuronal correlates of reappraisal in female patients with borderline personality disorder. Biological Psychiatry: Cognitive Neuroscience and Neuroimaging..

[30] Yehuda R, Hoge CW, McFarlane AC, Vermetten E, Lanius RA, Nievergelt CM (2015). Post-traumatic stress disorder. Nat Rev Dis Primers.

[31] Gunderson JG, Herpertz SC, Skodol AE, Torgersen S, Zanarini MC (2018). Borderline personality disorder. Nat Rev Dis Primers..

[32] Minzenberg MJ, Fan J, New AS, Tang CY, Siever LJ (2007). Fronto-limbic dysfunction in response to facial emotion in borderline personality disorder: an event-related fMRI study. Psychiatry Res Neuroimaging.

[33] Uddin LQ, Yeo BTT, Spreng RN (2019). Towards a universal taxonomy of macro-scale functional human brain networks. Brain Topogr.

[34] Fassbinder E, Schweiger U, Martius D, Brand-de Wilde O, Arntz A. Emotion Regulation in Schema Therapy and Dialectical Behavior Therapy. Frontiers in Psychology. 2016;7(1373). 10.3389/fpsyg.2016.01373 PMC5021701 27683567

[35] Akiki TJ, Averill CL, Abdallah CG (2017). A network-based neurobiological model of PTSD: evidence from structural and functional neuroimaging studies. Curr Psychiatry Rep.

[36] Koch SB, van Zuiden M, Nawijn L, Frijling JL, Veltman DJ, Olff M (2016). Aberrant resting-state brain activity in posttraumatic stress disorder: a meta-analysis and systematic review. Depress Anxiety.

[37] Wang T, Liu J, Zhang J, Zhan W, Li L, Wu M (2016). Altered resting-state functional activity in posttraumatic stress disorder: a quantitative meta-analysis. Sci Rep.

[38] Wolf RC, Sambataro F, Vasic N, Schmid M, Thomann PA, Bienentreu SD, Wolf ND (2011). Aberrant connectivity of resting-state networks in borderline personality disorder. Journal of psychiatry & neuroscience: JPN.

[39] Lanius RA, Vermetten E, Loewenstein RJ, Brand B, Schmahl C, Bremner JD, Spiegel D (2010). Emotion modulation in PTSD: clinical and neurobiological evidence for a dissociative subtype. Am J Psychiatr.

[40] Dewar M, Paradis A, Fortin CA (2020). Identifying trajectories and predictors of response to psychotherapy for post-traumatic stress disorder in adults: a systematic review of literature. Can J Psychiatry.

[41] Barawi KS, Lewis C, Simon N, Bisson JI (2020). A systematic review of factors associated with outcome of psychological treatments for post-traumatic stress disorder. Eur J Psychotraumatol.

[42] Yuan M, Qiu C, Meng Y, Ren Z, Yuan C, Li Y, et al. Pre-treatment resting-state functional MR imaging predicts the long-term clinical outcome after short-term Paroxtine treatment in post-traumatic stress disorder. Frontiers in psychiatry. 2018;9. 10.3389/fpsyt.2018.00532. 10.3389/fpsyt.2018.00532 PMC6218594 30425661

[43] Etkin A, Maron-Katz A, Wu W, Fonzo GA, Huemer J, Vértes PE (2019). Using fMRI connectivity to define a treatment-resistant form of post-traumatic stress disorder. Science Translational Med.

[44] Zhutovsky P, Thomas RM, Olff M, van Rooij SJH, Kennis M, van Wingen GA, et al. Individual Prediction of Psychotherapy Outcome in Posttraumatic Stress Disorder using Neuroimaging Data. bioRxiv. 2019:647925. 10.1038/s41398-019-0663-7 PMC6889413 31792202

[45] Bryant R, Felmingham K, Kemp A, Das P, Hughes G, Peduto A (2008). Amygdala and ventral anterior cingulate activation predicts treatment response to cognitive behaviour therapy for post-traumatic stress disorder. Psychol Med.

[46] Van Rooij SJ, Kennis M, Vink M, Geuze E (2016). Predicting treatment outcome in PTSD: a longitudinal functional MRI study on trauma-unrelated emotional processing. Neuropsychopharmacology..

[47] Cisler JM, Sigel BA, Kramer TL, Smitherman S, Vanderzee K, Pemberton J, Kilts CD (2015). Amygdala response predicts trajectory of symptom reduction during trauma-focused cognitive-behavioral therapy among adolescent girls with PTSD. J Psychiatr Res.

[48] Aupperle RL, Allard CB, Simmons AN, Flagan T, Thorp SR, Norman SB, Paulus MP, Stein MB (2013). Neural responses during emotional processing before and after cognitive trauma therapy for battered women. Psychiatry Research - Neuroimaging.

[49] Falconer E, Allen A (2013). Felmingham KL.

[50] Van Rooij S, Kennis M, Sjouwerman R, Van Den Heuvel M, Kahn R, Geuze E (2015). Smaller hippocampal volume as a vulnerability factor for the persistence of post-traumatic stress disorder. Psychol Med.

[51] Bryant RA, Felmingham K, Whitford TJ, Kemp A, Hughes G, Peduto A, Williams LM (2008). Rostral anterior cingulate volume predicts treatment response to cognitive-behavioural therapy for posttraumatic stress disorder. J Psychiatry Neuroscience.

[52] Schmitgen MM, Niedtfeld I, Schmitt R, Mancke F, Winter D, Schmahl C, et al. Individualized treatment response prediction of dialectical behavior therapy for borderline personality disorder using multimodal magnetic resonance imaging. Brain and behavior. 2019:e01384. 10.1002/brb3.1384 PMC6749487 31414575

[53] Effectiveness of PTSD-treatment Compared to Integrated PTSD-PD-treatment in Adult Patients With Comorbid PTSD and BPD [Available from: https://ClinicalTrials.gov/show/NCT03833453.

[54] Effectiveness of PTSD-treatment Compared to Integrated PTSD-PD-treatment in Adult Patients With Comorbid PTSD and CPD [Available from: https://ClinicalTrials.gov/show/NCT03833531.

[55] Patel R, Spreng RN, Shin LM, Girard TA (2012). Neurocircuitry models of posttraumatic stress disorder and beyond: a meta-analysis of functional neuroimaging studies. Neurosci Biobehav Rev.

[56] Rauch SL, Shin LM, Phelps EA (2006). Neurocircuitry models of posttraumatic stress disorder and extinction: human neuroimaging research—past, present, and future. Biol Psychiatry.

[57] First MW, JBW; Benjamin, LS; Spitzer, RL. (2015). User's guide for the SCID-5-PD (structured clinical interview for DSM-5 personality disorder).

[58] Thirion B, Pinel P, Mériaux S, Roche A, Dehaene S, Poline J-B (2007). Analysis of a large fMRI cohort: statistical and methodological issues for group analyses. Neuroimage..

[59] Desmond JE, Glover GH (2002). Estimating sample size in functional MRI (fMRI) neuroimaging studies: statistical power analyses. J Neurosci Methods.

[60] Frijling JL, van Zuiden M, Koch SB, Nawijn L, Veltman DJ, Olff M (2016). Effects of intranasal oxytocin on amygdala reactivity to emotional faces in recently trauma-exposed individuals. Soc Cogn Affect Neurosci.

[61] Snoek A, Beekman ATF, Dekker J, Aarts I, van Grootheest G, Blankers M, Vriend C, van den Heuvel O, Thomaes K (2020). A randomized controlled trial comparing the clinical efficacy and cost-effectiveness of eye movement desensitization and reprocessing (EMDR) and integrated EMDR-dialectical Behavioural therapy (DBT) in the treatment of patients with post-traumatic stress disorder and comorbid (sub) clinical borderline personality disorder: study design. BMC Psychiatry.

[62] van den End A, Dekker J, Beekman ATF, Aarts I, Snoek A, Blankers M (2021). Clinical Efficacy and Cost-Effectiveness of Imagery Rescripting Only Compared to Imagery Rescripting and Schema Therapy in Adult Patients With PTSD and Comorbid Cluster C Personality Disorder: Study Design of a Randomized Controlled Trial. Frontiers in psychiatry.

[63] Weiner MW, Veitch DP, Aisen PS, Beckett LA, Cairns NJ, Green RC, Harvey D, Jack CR, Jagust W, Morris JC, Petersen RC, Salazar J, Saykin AJ, Shaw LM, Toga AW, Trojanowski JQ, Alzheimer's Disease Neuroimaging Initiative (2017). The Alzheimer's disease neuroimaging initiative 3: continued innovation for clinical trial improvement. Alzheimers Dement.

[64] Langner O, Dotsch R, Bijlstra G, Wigboldus DH, Hawk ST, Van Knippenberg A (2010). Presentation and validation of the Radboud faces database. Cognit Emot.

[65] Smith SM, Jenkinson M, Woolrich MW, Beckmann CF, Behrens TE, Johansen-Berg H (2004). Advances in functional and structural MR image analysis and implementation as FSL. Neuroimage..

[66] Andersson JL, Skare S, Ashburner J (2003). How to correct susceptibility distortions in spin-echo echo-planar images: application to diffusion tensor imaging. Neuroimage..

[67] Esteban O, Birman D, Schaer M, Koyejo OO, Poldrack RA, Gorgolewski KJ (2017). MRIQC: Advancing the automatic prediction of image quality in MRI from unseen sites. PloS one.

[68] Power JD, Schlaggar BL, Petersen SE (2015). Recent progress and outstanding issues in motion correction in resting state fMRI. NeuroImage..

[69] Esteban O, Markiewicz CJ, Blair RW, Moodie CA, Isik AI, Erramuzpe A, Kent JD, Goncalves M, DuPre E, Snyder M, Oya H, Ghosh SS, Wright J, Durnez J, Poldrack RA, Gorgolewski KJ (2019). fMRIPrep: a robust preprocessing pipeline for functional MRI. Nat Methods.

[70] Andersson JLR, Sotiropoulos SN (2016). An integrated approach to correction for off-resonance effects and subject movement in diffusion MR imaging. NeuroImage..

[71] Tournier JD, Smith R, Raffelt D, Tabbara R, Dhollander T, Pietsch M, Christiaens D, Jeurissen B, Yeh CH, Connelly A (2019). MRtrix3: a fast, flexible and open software framework for medical image processing and visualisation. NeuroImage..

[72] Zhang H, Yushkevich PA, Alexander DC, Gee JC (2006). Deformable registration of diffusion tensor MR images with explicit orientation optimization. Med Image Anal.

[73] OSF. Open Science Framework [Available from: www.osf.io.

[74] Desikan RS, Ségonne F, Fischl B, Quinn BT, Dickerson BC, Blacker D, Buckner RL, Dale AM, Maguire RP, Hyman BT, Albert MS, Killiany RJ (2006). An automated labeling system for subdividing the human cerebral cortex on MRI scans into gyral based regions of interest. NeuroImage..

[75] Fan L, Li H, Zhuo J, Zhang Y, Wang J, Chen L, Yang Z, Chu C, Xie S, Laird AR, Fox PT, Eickhoff SB, Yu C, Jiang T (2016). The human Brainnetome atlas: a New brain atlas based on connectional architecture. Cereb Cortex.

[76] Schaefer A, Kong R, Gordon EM, TO L, Zuo X-N, Holmes AJ (2017). Local-global Parcellation of the human cerebral cortex from intrinsic functional connectivity MRI. Cereb Cortex.

[77] Rubinov M, Sporns O (2010). Complex network measures of brain connectivity: uses and interpretations. Neuroimage..

[78] Sizemore AE, Bassett DS (2018). Dynamic graph metrics: tutorial, toolbox, and tale. NeuroImage..

[79] McLaren DG, Ries ML, Xu G, Johnson SC (2012). A generalized form of context-dependent psychophysiological interactions (gPPI): a comparison to standard approaches. Neuroimage..

[80] Weathers F, Blake D, Schnurr P, Kaloupek D, Marx B, Keane T (2015). The Clinician-Administered PTSD Scale for DSM-5 (CAPS-5)–past month.

[81] Jacobson NS, Truax P (1992). Clinical significance: a statistical approach to defining meaningful change in psychotherapy research.

[82] Gratz KL, Roemer L (2004). Multidimensional assessment of emotion regulation and dysregulation: development, factor structure, and initial validation of the difficulties in emotion regulation scale. J Psychopathol Behav Assess.

[83] Rytwinski NK, Scur MD, Feeny NC, Youngstrom EA (2013). The co-occurrence of major depressive disorder among individuals with posttraumatic stress disorder: a meta-analysis. J Trauma Stress.

[84] Schulze L, Schulze A, Renneberg B, Schmahl C, Niedtfeld I (2019). Neural correlates of affective disturbances: a comparative meta-analysis of negative affect processing in borderline personality disorder, major depressive disorder, and posttraumatic stress disorder. Biological Psychiatry: Cognitive Neuroscience and Neuroimaging.

[85] Hutsebaut J, Willemsen E, Van H (2018). Time for cluster C personality disorders: state of the art. Tijdschrift voor psychiatrie.

