# Supplementary material for: Neural correlates of treatment effect and prediction of treatment outcome in patients with PTSD and comorbid personality disorder: study design
Source: Borderline Personal Disord Emot Dysregul. 2021 May 5;8:13. doi: 10.1186/s40479-021-00156-8 (PMC8097786; doi:10.1186/s40479-021-00156-8)
Supplement: Supplementary file 1 — Additional file 1 Table S1. Overview of models used in the Emotional Faces task. [file 40479_2021_156_MOESM1_ESM.docx]

**SUPPLEMENTS**

Supplementary Table 1: Overview of models used in the Emotional Faces task

|  | Version 1 | Version 2 |
| --- | --- | --- |
| Male models | 3, 5, 7, 9, 19, 15, 20, 21 | 24, 25, 33, 38, 46, 47, 49, 71 |
| Female models | 1, 2, 4, 8, 12, 14, 16, 18 | 22, 27, 31, 37, 56, 57, 58, 61 |

*Overview of models in the Emotional Faces Task, from the Radboud Faces Database*
